# Abnormal surface morphology of the central sulcus in children with attention-deficit/hyperactivity disorder

**DOI:** 10.3389/fnana.2015.00114

**Published:** 2015-08-28

**Authors:** Shuyu Li, Shaoyi Wang, Xinwei Li, Qiongling Li, Xiaobo Li

**Affiliations:** ^1^School of Biological Science and Medical Engineering, Beihang UniversityBeijing, China; ^2^Department of Biomedical Engineering, New Jersey Institute of TechnologyNewark, NJ, USA; ^3^The Gruss Magnetic Resonance Research Center, Department of Radiology, Albert Einstein College of MedicineNew York, NY, USA

**Keywords:** central sulcus, ADHD, surface morphology, MRI

## Abstract

The central sulcus (CS) divides the primary motor and somatosensory areas, and its three-dimensional (3D) anatomy reveals the structural changes of the sensorimotor regions. Attention-deficit/hyperactivity disorder (ADHD) is a neurodevelopmental disorder that is associated with sensorimotor and executive function deficits. However, it is largely unknown whether the morphology of the CS alters due to inappropriate development in the ADHD brain. Here, we employed the sulcus-based morphometry approach to investigate the 3D morphology of the CS in 42 children whose ages spanned from 8.8 to 13.5 years (21 with ADHD and 21 controls). After automatic labeling of each CS, we computed seven regional shape metrics for each CS, including the global average length, average depth, maximum depth, average span, surface area, average cortical thickness, and local sulcal profile. We found that the average depth and maximum depth of the left CS as well as the average cortical thickness of bilateral CS in the ADHD group were significantly larger than those in the healthy children. Moreover, significant between-group differences in the sulcal profile had been found in middle sections of the CSs bilaterally, and these changes were positively correlated with the hyperactivity-impulsivity scores in the children with ADHD. Altogether, our results provide evidence for the abnormity of the CS anatomical morphology in children with ADHD due to the structural changes in the motor cortex, which significantly contribute to the clinical symptomatology of the disorder.

## Introduction

The central sulcus (CS) is one of the most prominent and stable sulci of the human brain, which divides the primary motor and somatosensory areas. The three-dimentional (3D) anatomy of the CS includes motor and sensory maps, somatotopically organized according to Penfield’s (1937) classical ‘homunculus.’ Investigation of the 3D sulcal anatomy of the CS could reveal characteristic morphological features and further explore the associations between anatomy and function (Sastre-Janer et al., 1998). Many studies have reported that the morphological characteristics of the CS can be affected by factors such as handedness (Sun et al., 2012), learning (Li et al., 2010), gender (Cykowski et al., 2008), normal aging (Li et al., 2011), and neuropsychiatric disorders (Fujiwara et al., 2007).

Attention-Deficit/Hyperactivity Disorder (ADHD) is one of the most common childhood-onset neuropsychiatric disorders, characterized by hyperactivity, impulsivity, inattention, or a combination (First, 1994). Deficits of motor functions in children with ADHD have been frequently reported, such as slower speed (Denckla and Rudel, 1978) and greater degree of motor overflow movements (Mostofsky et al., 2003) when performing timed motor tasks, compared with typically developing controls. Previous functional neuroimaging studies have demonstrated dysfunctional neural activity of the motor cortex in ADHD children, such as reduced cortical activity and spatial extent of activation in the primary motor and parietal cortices (Mostofsky et al., 2006). Meanwhile, structural MRI studies in children with ADHD have reported atypical brain morphology in sensorimotor brain regions, such as gray matter (GM) volume decreases in the left precentral and postcentral areas (Carmona et al., 2005), and cortical thickness reductions of the precentral cortex (Shaw et al., 2006; Narr et al., 2009; Hoekzema et al., 2012). However, the morphological characteristics of the CS in children with ADHD and their relationships with the clinical symptoms have not yet been investigated.

Surface-based approaches provide a framework for identifying local changes across the surface anatomy of the sulcus. This is accomplished using 3D surface parameterization following automated sulcal mesh reconstruction, which would enable further shape analysis, such as the encoding of statistical properties in local anatomical variations within individual sulci. Sulcal parameterization is a critical step, and it can create a normalized coordinate system on the CS surfaces that allows to compare the morphological differences at each location of the CS across individuals. The method based on the heat-equation diffusion process along the CS surface (Cykowski et al., 2008) has been applied in sulcal parameterization (Coulon et al., 2011; McKay et al., 2013; Leroy et al., 2015). In addition, Large Diffeomorphic Deformation Mapping Metric (LDDMM; Trouve, 1998), as a powerful surface mapping method, can perform two cortical surfaces mapping by treating the two-dimensional manifolds of the cortical surfaces as one-dimensional features (curves), or two-dimensional structure of the manifold as a whole, or combination of one- and two-dimensional features (curves and surface; Glaunès et al., 2008; Zhong et al., 2010).

Here, we utilized a surface-based computational approach to investigate the 3D morphology of the CSs in children with ADHD. We first extracted the CS from individual, high-resolution structural MR images, followed by 3D surface reconstruction and parameterization. We then computed several surface metrics (sulcal length, depth, surface area, sulcal span, cortical thickness, and sulcal profile) based on the parameterized surface maps. Finally, we statistically analyzed the differences between the children with ADHD and the controls with regard to these metrics.

## Materials and Methods

### Participants

The sample for this study consisted of 21 children with ADHD and 21 demographically group-matched control children, ages from 9 to 15 years old. All of the subjects were strongly right-handed, evaluated using the Edinburgh Handedness Inventory (Oldfield, 1971) and had estimated full-scale IQ ≥ 80, measured by Wechsler Abbreviated Scale of Intelligence (WASI; Wechsler, 1999), to minimize neurobiological heterogeneity.

The patient group included children who met *DSM-IV* criteria for ADHD combined type, by combining the Conners Rating Scale—Revised-L for both parent and self-reports (Conners, 1997). This was confirmed with a parent interview using the Schedule for Affective Disorders and Schizophrenia for Children—Present and Lifetime Version (K-SADS-PL; Kaufman et al., 1997). The normal control group included children who had *T*-scores <60 (<1 SD) on all Conners parent and self-reports. For both groups, we included the K-SADS-PL screening questions and supplements to rule out pervasive developmental disorders, substance use and abuse, and posttraumatic stress disorder. Similarly, oppositional defiant disorder with physical aggression (using DSM-IV diagnostic criteria), and all other current Axis I disorders (except for fear of the dark) were exclusionary. Children with any specific learning disorders were also excluded. The basic reading, mathematical reasoning, reading comprehension, and numerical operations subtests of the Wechsler Individual Achievement Test Second Edition (WIAT-II; Wechsler, 2001) were administered to determine the presence of impairments in reading or math. General exclusion criteria for both groups also included chronic medical/neurological illness or was taking systemic medication; specific or focal neurological disorder including epilepsy; treatment with any non-stimulant psychotropic within the past 3 months; and contraindications to magnetic resonance imaging (MRI) scanning. In addition, we also avoided including siblings, considering that genetic factors might influence the morphological patterns of the CS.

The children with ADHD were recruited from the Children’s Evaluation and Rehabilitation Center at the Albert Einstein College of Medicine, and the Max and Celia Parnes Family Psychological and Psycho-educational Services Clinic at the Ferkauf Graduate School of Psychology. The control children were recruited from local schools through newspaper advertisements. This study received Institutional Review Board approval for human subjects’ research at the Albert Einstein College of Medicine. Written informed consents were provided by all participants and their parents after the nature of the study and its procedures were carefully explained. All procedures were conducted in keeping with the tenets for the ethical conduct of research as outlined in the Declaration of Helsinki.

### MRI Scan Acquisition and Image Preprocessing

**FIGURE 1:**
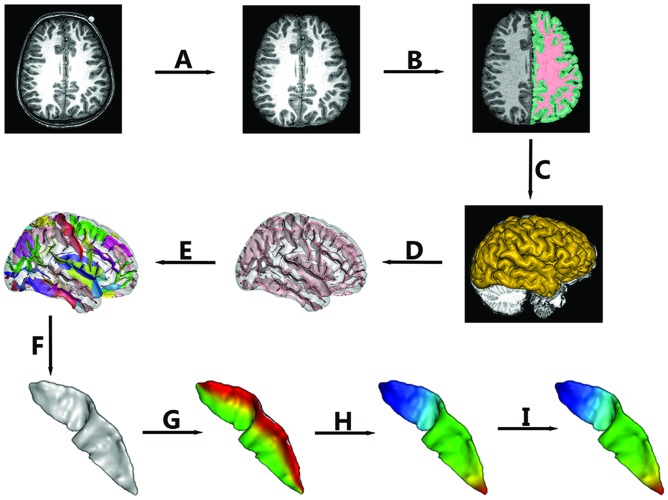
**The pipeline of central sulcus (CS) extraction and parameterization.** Extraction of CS consisted of the following steps: **(A)** Skull stripping; **(B)** Spatial normalization, RF homogeneity correction, and tissue segmentation; **(C)** Extraction of gray matter (GM)/cerebrospinal fluid (CSF) surfaces; **(D)** Extraction of sulcal folds; **(E)** Automated labeling of sulci; **(F)** Manual verification of the CS. Sulcal parameterization included three steps: **(G)** Parameterization along the *x* coordinate; **(H)** Parameterization along the *y* coordinate; **(I)** Reparameterization by rescaling the *y* coordinate.

High-resolution three-dimensional T1-weighted structural MRI data were acquired from each subject using a 3T Philips Achiva TX MR system with a 32-channel phased array head coil (Invivo, Gainesville, FL, USA). Axial images were acquired using MPRAGE with the following scan parameters: TR = 9.8 ms, TE = 4.6 ms, flip angle = 8°, voxel size = 0.94 mm × 0.94 mm × 1 mm, field of view = 240 mm × 188 mm × 220 mm, SENSE reduction factor = 2.5, 235 contiguous slices. Then, all images were resliced at a 0.94 mm × 0.94 mm × 0.94 mm isotropic resolution. The non-brain tissue was stripped (See **Figure 1A**) using the Brain Extraction Tool (Smith, 2002) provided as an add-on for the MRIcro software package^1^ (FMRIB Image Analysis Group, Oxford, UK).

### Extraction of the CS

In this study, BrainVISA (BV) software^2^ (version 4.3.0) was used to extract the CS for all of the individuals. The whole pipeline is shown in **Figure 1**. The detailed procedures were as follows.

#### (a) Normalization

To remove gross differences in brain size and orientation, we normalized all images into Talairach space. First, two professional neuroanatomists manually labeled four points in each axial image, i.e., the anterior commissure, the posterior commissure, an inter-hemispheric point, and a point in the left hemisphere. Then, a transformation based on the two points defined by the anterior and posterior commissures to the Talairach AC/PC referential was computed and applied to the whole image.

#### (b) Brain Tissue Segmentation

The intensities in raw images not only depend on the properties of different tissues but also on the location in the field of view due to the inhomogeneity of magnetic field. Thus, we first corrected the spatial bias (Mangin, 2000) in normalized images. Then, a fuzzy-classifier-based anatomical segmentation method (Mangin et al., 1998) was used to segment brain tissue into GM, white matter (WM), and cerebrospinal fluid (CSF; See **Figure 1B**).

#### (c) Sulcal Extraction and Identification of the CS

A 3D mesh of the external surface of the cortex for each hemisphere was computed based on the boundary between GM and CSF, as shown in **Figure 1C**. Sulcal structures were then reconstructed as the medial surfaces of two opposing gyral banks, which spanned from the GM/CSF border at the most internal point of the sulcus to the convex hull of the cortex (See **Figure 1D** and Mangin et al., 2004). Finally, an automated sulcal recognition algorithm was used to assign standard anatomical labels to these sulci, including the CS (See **Figure 1E**).

#### (d) Manual Verification of the CS

We observed some small, accessory sulci on the surfaces of the extracted CSs. They were usually shorter than 5 mm, originating at a right angle from the stem of the main sulcus, and were considered to be random folds (Ono et al., 1990). In this study, two experienced neuroanatomists checked each extracted CS and removed these small and accessory sulci. An example of the finally extracted CS was shown in **Figure 1F**.

### Sulcal Parameterization

To compare the morphological differences at each location of the CS across individuals, we need to parameterize each CS to create a normalized coordinate system on the CS surfaces. Our sulcal parameterization included two steps. The first step was to acquire the *x–y* coordinate system for each CS using the sulcal parameterization method based on the heat-equation diffusion process along the CS surface (Cykowski et al., 2008). Two coordinate fields (*x*: from the lateral to the medial edge; and *y*: from the superior to the inferior end of the sulcus) were extrapolated by solving the heat equation on the surface, and the bottom and top ridges of the sulcal mesh as well as the endpoints of the sulcus where these top and bottom ridges joined behaved as constant heat sources (**Figures 1G,H**). In this way, the normalized coordinate system was constructed for each CS surface.

**FIGURE 2:**
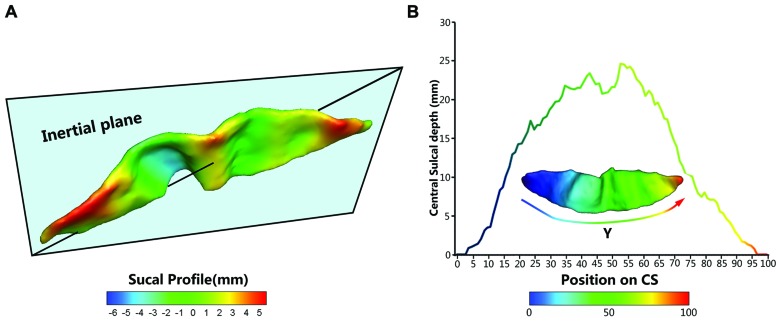
**The central sulcal profile and the Depth-Position Profile (DPP). (A)** Central sulcal profile coded by different colors (from negative in blue to positive in red) which is defined the signed distance between each vertex on the CS surface and inertia plane. **(B)** A CS mesh with *y* coordinate field and its depth curve which is defined as the distance between the paired points at the sulcal fundus and the brain envelope that shared the same *y* coordinate along the parameterized sulcal mesh surface.

To provide a better inter-subject matching of the CS anatomy, a reparameterization procedure based on two anatomical landmarks on the sulcal profile was performed as the second step. This method was similar to those used in this literature (Coulon et al., 2011). The sulcal profile was defined as the function of the *y* position that represented the average value of the signed distance of each node on the mesh at the same position *y* to the inertial plane of the CS. The inertial plane was defined by the barycenter and was reflected in two orthogonal principal orientations of the CS (See **Figure 2A**). The sulcal profile was a morphological characteristic to measure shape variations along the superior–inferior direction (i.e., the direction along the *y*-axis) of the CS (Coulon et al., 2011 and See **Figure 2A**). Then, two stable anatomical landmarks, L_1_ and L_2,_ relating to the functional primary motor area of the hand, were detected on the CS along the sulcal profile curve according to the literature (Coulon et al., 2011). For each group, the average L_1_ and L_2_ were obtained by averaging the L_1_ and L_2_ positions across subjects within the same group. The reparameterization was computed by rescaling the *y* coordinates in a piecewise linear fashion by exactly matching the coordinates of the two anatomical landmarks L_1_ and L_2_ across subjects within each group. This step ensured the correspondences of the surface morphology of the hand area in the CS across subjects, as depicted in **Figure 1I**. Finally, all parameterized CS meshes were remeshed to the template mesh to obtain an inter-subject node-to-node correspondence. The template CS mesh was constructed from an unbiased standard MRI template brain data within the 10-to-14-year-old age range (Almli et al., 2007).

### Surface-based Measurements

We employed five metrics (i.e., sulcal depth, length, span, surface area, and cortical thickness) to describe the entire morphometry of the CS surface. The *sulcal depth* at each position was defined as the distance between the paired points at the sulcal fundus and the brain envelope that shared the same *y* coordinate along the parameterized sulcal mesh surface (See **Figure 2B**). The average sulcal depth was calculated as the mean value of the sulcal depths at all positions. The maximum depth was also chosen from all sulcal depths for each subject. The *sulcal length* was defined as the average length along the exterior and interior sulcal boundaries of CS. The average *sulcal span* for the CS was defined as an average 3D distance between opposing gyral banks along the normal directions of the medial sulcal mesh (Kochunov et al., 2008). The *sulcal surface area* was evaluated as the sum of the areas of all triangles of the mesh of the CS. The average *cortical thickness* was the mean distance between the CSF/GM interface and the GM/WM interface along the CS.

In addition, we used the signed distance of each node on the mesh to the inertial plane of the CS to characterize the local shape variations on the surface. The signed distance was defined as *d*_*i*_ = (n_*i*_ - 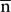) u_3_ where 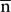 is the barycenter of the CS mesh and n_*i*_ = (*n*_*xi*_, *n*_*yi*_, *n*_*zi*_)^*T*^ represents the node of the mesh and where a given sulcus mesh could be defined as the vector of nodes S = (n_*i*_)_*i* = 1....*N*_. The centered mesh S_*c*_ was defined as (n_*i*_ - 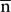). We generated the covariance matrix $A\,=S_{c}S_{c}^{T}$, and u_1_, u_2_, and u_3_ are the three eigenvectors of A ordered by decreasing eigen-value. Thus, u_1_ and u_2_ represent the main orientation of the CS and construct the inertia plane, and u_3_ reflects the normal direction of the inertial plane (Coulon et al., 2011).

### Statistical Analysis

For each sulcal metric, we used a multiple linear regression model to explore the between-group differences, with age and gender as covariates, because the 3D morphology of the CS could be affected by age and gender (Amunts et al., 2000; Luders et al., 2003). *P*-values less than 0.05 were considered to be statistically significant.

To determine whether the CS depth and profile in the children with ADHD were different along the superior-inferior direction (i.e., the *y*-axis) of the CS compared with controls, we performed multiple linear regression analyses at each *y* position with age and gender as the covariates. Significant differences were evident in the sulcal depth and profile curves. To further estimate the local shape variations on the surface, we compared the between-group differences in the signed distance on the mesh to the inertial plane in a node-by-node manner. Statistical difference maps for the sulcal profiles between the groups are displayed on the template. The false discovery rate (FDR) was used for multiple-comparison corrections.

Furthermore, we assessed the associations between these sulcal metrics and hyperactivity–impulsivity scores as well as inattention scores in the regions showing significant between-group differences across all subjects, using age, and gender as the covariates.

## Results

### Group Statistics

The children with ADHD and typically developing controls had no significant differences in demographic measures (all *P* > 0.1). The *t*-scores of inattention, hyperactivity–impulsivity, and DSM-IV total in ADHD children were significantly higher when compared to that in controls. Detailed statistics are shown in **Table 1**.

**Table 1:** Demographic and clinical data of the healthy children and the children with attention-deficit/hyperactivity disorder (ADHD).

	ADHD (*n* = 21) Mean (SD)	Healthy (*n* = 21) Mean (SD)	*P*
Age (months)	130.14 (23.83)	139.19 (22.71)	0.2152
Gender (boys/girls)	18/3	15/6	0.1571
Inattention score^∗∗^	64.52 (9.27)	46.95 (5.63)	<<0.001
Hyperactivity-impulsivity scores^∗∗^	71.67 (14.00)	50.00 (10.93)	<<0.001
DSM-IV total scores^∗∗^	69.19 (10.68)	48.40 (8.22)	<<0.001
IQ	104.90 (15.61)	107.38 (12.10)	0.5688

*Inattention score, Hyperactivity–impulsivity scores, and DSM-IV total scores reflect DSM-IV criteria for ADHD. The threshold value is 60. A Wilcoxon rank-sum test and a two-sample t-test were separately used to examine differences in the gender and age distributions between the ADHD and control groups. ^∗∗^P < 0.001*.

### Between-Group Differences in Global Measures of the CS

Results of statistic analyses in the global measures (average depth, maximum depth, average length, average span, surface area, and average cortical thickness) of the CS are summarized in **Table 2**. The ADHD group showed a significantly greater sulcal depth (average depth: *P* < 0.01; maximum depth: *P* = 0.018) in the left CS compared to the control group. The average cortical thicknesses along the bilateral CS of the ADHD group were significantly larger than those of the control group (left: *P* < 0.01; right: *P* < 0.01).

**Table 2:** The global measures of the central sulcus (CS; Means ± SD).

	ADHD	Controls	*P*-value	*t*-value
**Average length (mm)**				
*Left*	99.31 ± 10.14	97.97 ± 8.41	0.619	0.502
*Right*	97.29 ± 7.61	94.89 ± 7.17	0.406	0.840
**Average depth (mm)**
*Left*	14.33 ± 1.28	13.00 ± 1.08	**0.003**^∗∗^	3.173
*Right*	14.64 ± 1.65	13.82 ± 0.97	0.074	1.837
**Maximum depth (mm)**				
*Left*	19.42 ± 1.49	18.25 ± 1.31	**0.018**^∗∗^	2.470
*Right*	19.66 ± 1.91	18.53 ± 1.59	0.056	1.968
**Average span (mm)**				
*Left*	2.35 ± 0.16	2.43 ± 0.19	0.275	–1.106
*Right*	2.35 ± 0.15	2.29 ± 0.15	0.398	0.855
**Surface area (×10^3^ mm^2^)**				
*Left*	3.56 ± 0.45	3.47 ± 0.38	0.460	0.747
*Right*	3.52 ± 0.49	3.45 ± 0.80	0.843	0.120
**Average cortical thickness(mm)**				
*Left*	3.83 ± 0.33	3.23 ± 0.59	**0.0005**^∗∗^	3.83
*Right*	3.78 ± 0.37	3.13 ± 0.59	**0.0003**^∗∗^	3.99

*t > 0 represents the ADHD were larger than the controls.*

*^∗∗^P < 0.01.*

### Sulcal Depth and Sulcal Profile along the CS

**FIGURE 3:**
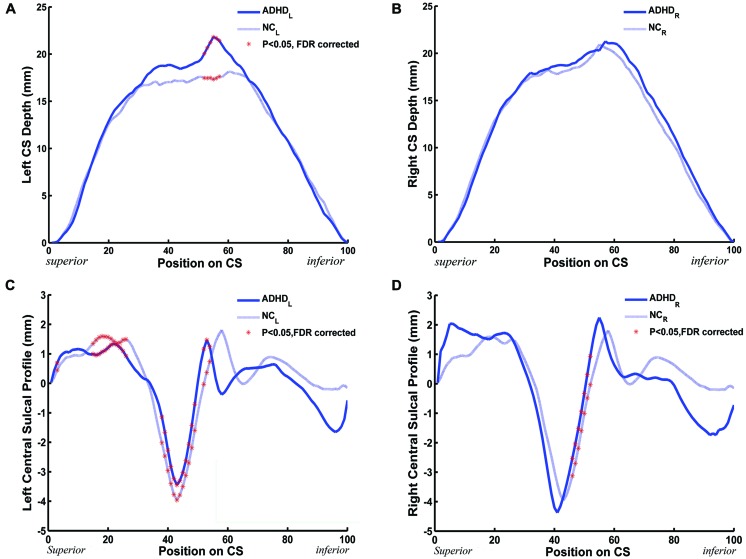
**The between-group (ADHD vs. NC) difference along the mean DPPs and the central sulcal profile curve. (A,B)** The mean DPP across the children with ADHD (blue solid trace) and the mean DPP across the healthy children (blue dashed trace) were plotted by position. **(C,D)** The average central sulcal profile of the children with ADHD (blue solid trace) and the average central sulcal profile of the healthy children (blue dashed trace) were plotted by position. The red stars represent the positions on the CS where the depth (left: 52 ≤ *y* ≤ 57; right: none) or the sulcal profile (left: *y* = 3,15 ≤ *y* ≤ 26, 38 ≤ *y* ≤ 49, 52 ≤ *y* ≤ 54; right: 46 ≤ *y* ≤ 52) of the CSs for the children with ADHD were significantly [^∗^*P*s < 0.05 after false discovery rate (FDR) correction] different from those of the healthy children.

We measured the CS depth along the *y* coordinate at 100 successive points and then produced the depth-position profile (DPP) after sulcal parameterization. The between-group differences of the local CS depth, along the superior-inferior direction of the bilateral CS, are shown in the **Figures 3A,B**. *P*s less than 0.05 were shown as red asterisks after FDR correction. Between group comparison of this local measure found that children with ADHD had significantly greater local depth in the middle section (i.e., *y* = 52∼57) of left side CS.

A similar analysis was applied to the sulcal profile. Scatter plots are shown in **Figures 3C,D**. The two clinical groups showed significant (*P* < 0.05, FDR corrected) differences of local CS profiles in large areas of the superior and middle sections (*y* = 15∼26, 38∼49, and 52∼54) of the left side CS, and in the middle section (*y* = 46∼52) of the right side CS.

### Node-based Analysis in the Sulcal Profile of the CS

**FIGURE 4:**
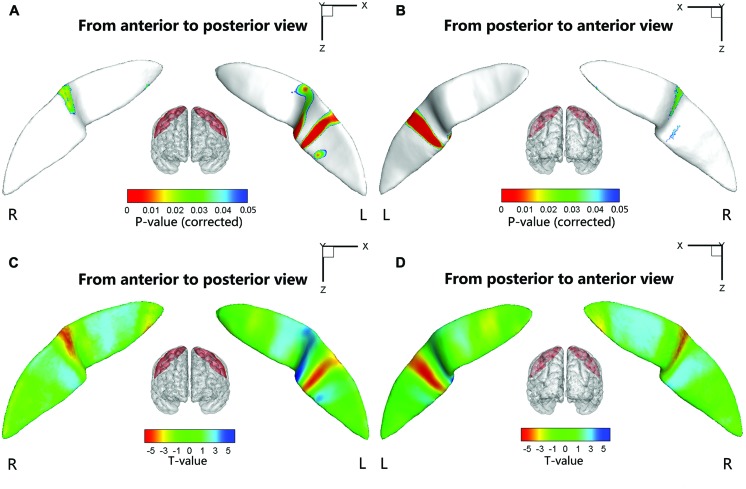
**Statistical differences of the node-based sulcal profile between the children with ADHD and the healthy children.** Compared with the healthy children, the children with ADHD showed significantly (*P*s < 0.05 after FDR correction; *t* < 0) nearer to the inertial plane (i.e., flat) in the middle section (i.e., somatotopic hand area; the direction of the sulcal profile in this area was positive on both sides of the CS) of the right CS. The anterior side of the left CS middle section also showed between-group differences. The upper-half part of this area in the children with ADHD was nearer to the inertia plane (*P* < 0.05 after FDR correction; *t* > 0; the direction of the sulcal profile in this area was positive), whereas the lower-half part was farther away from the inertial plane (*P* < 0.05 after FDR correction; *t* > 0; the direction of the sulcal profile in this area was negative). For the lower section of the left CS, the children with ADHD manifested greater closeness to the inertial plane on the anterior side of a strip area (*P* < 0.05 after FDR correction; *t* < 0; the direction of the sulcal profile in this area was positive), whereas on the posterior side of the strip area, the children with ADHD manifested greater distance from the inertia plane (*P* < 0.05 after FDR correction; *t* < 0; the direction of the sulcal profile in this area was negative). For the round area on the anterior side of the left CS, the children with ADHD manifested greater distance from the inertial plane (*P* < 0.05 after FDR correction; *t* > 0; the direction of the sulcal profile in this area was positive) than the healthy children. The results were overlapped with the mean CS surface that was obtained by averaging the position vector of the CS from all the subjects. L, left hemisphere; R, right hemisphere. **(A,C)** Anterior to posterior views of the CS. **(B,D)** Posterior to anterior views of the CS.

We also performed a node-based analysis to detect between-group differences in the absolute value of the signed distance of each node to the inertial plane on the CS surface. We found that regional shape variations were located in the middle sections of the bilateral CS (**Figure 4**). The warm color indicated that these regions on the CS surface were much farther from the inertial plane (i.e., exhibited more complex morphology) in the ADHD group compared with the control group. Moreover, the left CS showed more differences than the right CS.

### Relationships between the Sulcal Metrics and Clinical Measures

**FIGURE 5:**
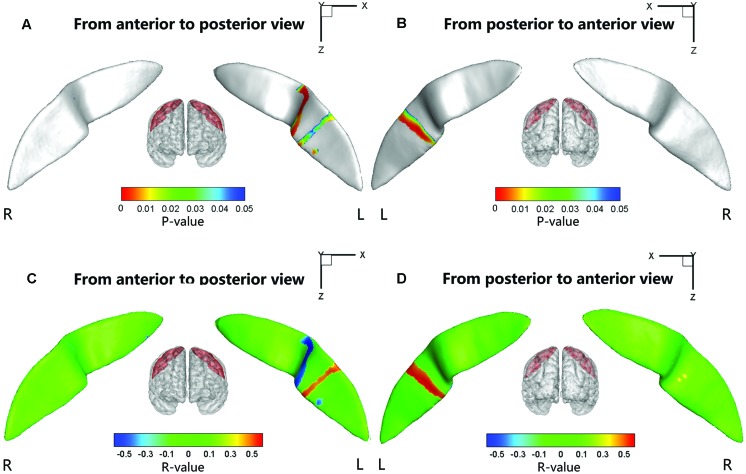
**The correlation between the node-based sulcal profiles and the hyperactivity-impulsivity scores using age and gender as the covariates.** The focal regions with significant positive correlations (*t* < 0, *P* < 0.05) were mapped onto the average surface of the CS. Notably, we first defined the regions showing significant differences in the sulcal profile between the two groups as a mask and then computed the correlation between the sulcal profile in each node within the mask and the hyperactivity–impulsivity scores of all subjects. L, left hemisphere; R, right hemisphere. **(A,C)** Anterior to posterior views of the CS. **(B,D)** Posterior to anterior views of the CS.

**FIGURE 6:**
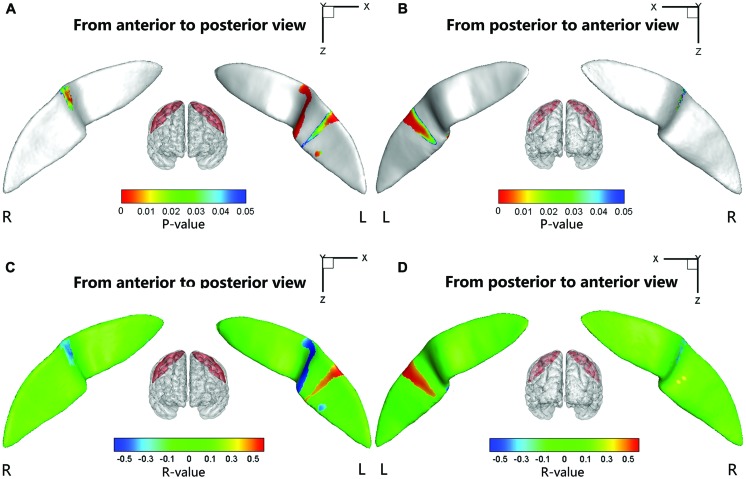
**The correlation between the node-based sulcal profiles and the inattention scores using age and gender as the covariates.** The focal regions with significant positive correlations (*t* < 0, *P* < 0.05) were mapped onto the average surface of the CS. Notably, we first defined the regions showing significant differences in the sulcal profile between the two groups as a mask and then computed the correlation between the sulcal profile in each node within the mask and the inattention scores of all subjects. L, left hemisphere; R, right hemisphere. **(A,C)** Anterior to posterior views of the CS. **(B,D)** Posterior to anterior views of the CS.

Significant positive associations between the cortical thickness along the bilateral CS and the inattention scores were found in the whole study sample (left: *P* = 0.0181; right: *P* = 0.0082), when using age and gender as the covariates. No significant associations among other global measures and hyperactivity-impulsivity scores as well as inattention scores were found in the whole study sample. For those regions showing significant between-group differences in node-based analysis, we further evaluated the relationships between the sulcal metrics and hyperactivity-impulsivity as well as inattention scores in the whole study sample. Significant (*P* < 0.05) negative correlations between the CS profile and the hyperactivity-impulsivity scores were found in several surface clusters on anterior side of the left CS, which were around the “hand knob” area, whereas the regions showing significant positive correlations located in the posterior side of left CS (anterior: **Figures 5A,C**); posterior: **Figures 5B,D**). Meanwhile, significant (*P* < 0.05) negative correlations between the CS profile and the inattention scores were found in clusters on anterior side of the left CS, while the regions showing positive correlations located in the posterior side of left CS (anterior: **Figures 6A,C**; posterior: **Figures 6B,D**).

## Discussion

In this study, we employed a sulcus-based computational approach to investigate the 3D morphology of the CS in children with ADHD. The morphological measures including the global average length, average depth, maximum depth, average span, surface area, and local sulcal profile were computed based on parameterized surface maps. Compared with healthy children, the children with ADHD had no significant differences in average length, average span, or surface area in the CS. However, we found that the children with ADHD showed significantly greater average and maximum depths of the left CS. Meanwhile, the between-group differences had been found in the sulcal profile of bilateral CS, and this change was associated with the hyperactivity–impulsivity scores and inattention scores of the ADHD children. Together, our results provided evidence for the abnormity of structural morphology of the CS in children with ADHD due to structural changes in the motor cortices of the brain.

### Morphometric Differences in the CS between the Children with ADHD and the Controls

We found that there were no significant differences in average length, average span and surface area of the bilateral CS between the children with ADHD and the healthy controls. However, the average and maximum depths of the left CS reflected a significantly deeper fold associated with ADHD compared with normal controls.

Sulcal depth has been widely used to characterize the morphology of cortical folding. Previous studies have suggested that changes in sulcal depth might result from altered axonal mechanical tension generated by cortico-cortical connections (Davatzikos and Bryan, 2002; Van Essen et al., 2006). Mostofsky et al. (2002) found that the premotor cortex of children with ADHD showed WM reduction on both sides, suggesting a primarily axonal abnormality in children with ADHD (Ranta et al., 2009). The maximum depth corresponds to the deepest part of a sulcus and was thought to be where the folding of the structure began (Smart and McSherry, 1986; Welker, 1990; Lefèvre et al., 2009); thus, it might be associated with geometrical features influenced by genetic factors. A substantial family (Biederman et al., 1990; Faraone et al., 1992), twin (Levy et al., 1997), and adoption studies (Alberts-Corush et al., 1986) have demonstrated genetic elements existing in ADHD. The heritability of ADHD has been estimated to be 0.50–0.98 (Levy et al., 1997). Meanwhile, the deep sulcal regions are thought to be the first cortical folds to form in the early stages of development (Régis et al., 2005), and their formation might be related to genetic control and cytoarchitectonic areas. Differences in depth might signal abnormal developmental events occurring in the early life stage, which would support the hypothesis of a departure from typical cortical developmental trajectory occurring very early in ADHD (Shaw et al., 2006).

Using DPPs, we further found that the depths at the positions (52∼57) of the left CS in the ADHD group were significantly larger than those of the typically developing children. These positions of the CS DPP are near the Sylvian fissure. Many published studies contributed to the mapping of the finger-tapping area (25∼47) of the DPP and to the mapping of the oral movement area, involving lip pursing, tongue movement, and smiling (57∼85), of the DPP (McKay et al., 2013). The abnormity in CS depth that is partly located on the oral movement area might explain the symptom of talkativeness in children with ADHD.

The sulcal profile curve is defined as the average signed distance of nodes that share the same position *y* to the inertial plane of the CS, which provides more shape information across subjects than the depth curve. In this study, we found that the between-group differences of the sulcal profile along the left CS were located in the middle section, which involved the function of finger tapping as well as oral movement. Subsequently, we further employed node-based analysis on the sulcal surface and located specific regions in the middle section of the bilateral CS showing significant between-group differences. Many studies have suggested that the middle section of the CS is mainly composed of the somatotopic hand area (White et al., 1997; Sastre-Janer et al., 1998; Boling et al., 1999). Functional neuroimaging studies have suggested that this region shows neuronal activation during hand motor tasks (Rao et al., 1995). Moll et al. (2001) used transcranial magnetic stimulation (TMS) to investigate the hand area of the left motor cortex and found a distinct dysfunctional pattern of deficient inhibitory motor control for ADHD and chronic tic disorder (TD), which could be a neurobiological correlate of hypermotoric symptoms in children with both disorders. Our findings of abnormities in the hand area of bilateral CS might account for the related ADHD deficits in executive and motor function. In addition, we also found that the lower section on both sides of the left CS showed significant between-group differences. Using functional MRI, Fesl et al. (2003) demonstrated that the inferolateral segment of the CS was mainly associated with the primary motor/sensory tongue area. These abnormities of the CS might explain the symptom of talkativeness in children with ADHD.

### Relationships between Sulcal Shape and the Gray Matter Volume, White Matter Volume, and Cortical Thickness of the Motor Area

Structural change in the motor area is of interest because motor hyperactivity is a cardinal feature of ADHD. Previous studies have reported the structural abnormality of the motor area in children with ADHD. The reduction of GM volume in the left perirolandic area (Carmona et al., 2005) or bilateral premotor areas (Mostofsky et al., 2002) were observed in children with ADHD, Shaw et al. (2007) observed cortical thickness maturational delays in ADHD and found that the maturational peak of the motor cortex in ADHD was 4 months ahead of the controls. They (Shaw et al., 2007) also found that children with ADHD manifested global thinning of the cortex, including the left precentral regions. We found the maximum and average depths of the left CS as well as average cortical thickness along bilateral CS in children with ADHD to be larger than those of the healthy children, whereas the other global sulcal metrics showed no between-group differences. A previous multiple regression analysis revealed that dilation of the sulcal space was related to reductions in the cortical GM thickness observed in normal aging (Kochunov et al., 2008). Im et al. (2008) suggest that there may be other effects that change the depth of the cortical sulci in normal subjects, i.e., severely reduced cortical thickness and gyral WM volume may overwhelm other effects and primarily lead to the sulcal depth changes. In our study, the increased sulcal depth and changes in the sulcal profile might be mostly caused by volumetric changes in the WM or GM and in cortical thickness, which can induce different biological and clinical interpretations and remains to be proved experimentally.

### Correlations between the CS Metrics and Clinical Measures

Very interestingly, this study found that in the whole study population, the average sulcal depth and node-based geometric properties of the anterior and posterior sides of the left CS (around the “hand knob” area) associated with the attention and inhibition capacities in similar ways. This finding first time placed the left CS into the brain networks (traditionally thought to involve prefrontal lobe, anterior cingulate cortex, striatum, thalamus, etc. (Castellanos et al., 2006) that may modulate the normality of attention and inhibition functions in a developmental brain, and contribute to the behavioral capacities of attention and inhibition.

## Further Considerations

To build upon this study, several issues need to be addressed. Firstly, it is not yet well known about how sulcal features directly relate to brain function and the functional implications of the findings need therefore to be explored in future studies. Secondly, we included both male and female subjects in both groups. We acknowledge possible gender-related differences of the GM and WM maturation patterns in typically developing children (Reiss et al., 1996; Eliez et al., 2001), and possible sex-related heterogeneity in ADHD (Ramtekkar et al., 2010). However, the sample size of our study was not large enough to run the between-gender comparisons in each group. Although we added gender as a fixed effect covariate for group comparisons, a future study should focus specifically on examining the gender effects upon abnormalities of the shape of the CS. Thirdly, there are some robust and straightforward shape morphometry analysis methods such as LDDMM (Trouve, 1998; Joshi and Miller, 2000) that can be used to investigate the CS morphology. In future studies, it would be meaningful to compare different surface analysis methods in characterizing sulcal geometry.

In summary, we presented a sulcal geometry-based statistical analysis approach to investigating the morphology of the CS in ADHD. Our study demonstrated significant morphological abnormalities in bilateral CS in children with ADHD, which were significantly associated with their clinical symptoms. These findings suggest that morphological alterations of the sensorimotor cortex in children with ADHD could significantly contribute to the anatomical substrates underlying clinical symptomatology of the disorder.

## Author Contributions

SL and XBL designed the research; XBL collected the data; SW, XWL, and QL analyzed the results; SL, SW, and XBL wrote the main manuscript text. All authors reviewed the manuscript.

## Conflict of Interest Statement

The authors declare that the research was conducted in the absence of any commercial or financial relationships that could be construed as a potential conflict of interest.

## Footnotes

1 http://www.sph.sc.edu/comd/rorden/mricro.html

2 http://brainvisa.info/
